# Comparison of endogenous and overexpressed MyoD shows enhanced binding of physiologically bound sites

**DOI:** 10.1186/2044-5040-3-8

**Published:** 2013-04-08

**Authors:** Zizhen Yao, Abraham P Fong, Yi Cao, Walter L Ruzzo, Robert C Gentleman, Stephen J Tapscott

**Affiliations:** 1 Human Biology Division, Seattle, WA, USA; 2 Clinical Research Division, Fred Hutchinson Cancer Research Center, 1100 Fairview Avenue North, Seattle, WA, 98109, USA; 3 Bioinformatics and Computational Biology, Genentech, South San Francisco, CA, USA; 4 Department of Pediatrics, University of Washington, School of Medicine, Seattle, WA, 98105, USA; 5 Departments of Computer Science and Engineering and Genome Sciences, Seattle, WA, USA; 6 Department of Neurology, University of Washington, School of Medicine, Seattle, WA, 98105, USA

**Keywords:** Transcription factor, Overexpressed, MyoD, c-Myc, ChIP-seq

## Abstract

**Background:**

Transcription factor overexpression is common in biological experiments and transcription factor amplification is associated with many cancers, yet few studies have directly compared the DNA-binding profiles of endogenous versus overexpressed transcription factors.

**Methods:**

We analyzed MyoD ChIP-seq data from C2C12 mouse myotubes, primary mouse myotubes, and mouse fibroblasts differentiated into muscle cells by overexpression of MyoD and compared the genome-wide binding profiles and binding site characteristics of endogenous and overexpressed MyoD.

**Results:**

Overexpressed MyoD bound to the same sites occupied by endogenous MyoD and possessed the same E-box sequence preference and co-factor site enrichments, and did not bind to new sites with distinct characteristics.

**Conclusions:**

Our data demonstrate a robust fidelity of transcription factor binding sites over a range of expression levels and that increased amounts of transcription factor increase the binding at physiologically bound sites.

## Background

The biological sciences have always relied on model systems to test specific hypotheses, and the general validity of each model system is constantly subject to vigorous debate. This is particularly true when transcription factors are overexpressed in cells, and the investigator(s) extrapolate their findings to the function of the endogenous factor. Although overexpression studies have yielded many significant advances in our understanding of cell biology, their validity is routinely challenged, particularly in manuscript and grant reviews. Intuitively this skepticism is justified. Biochemistry predicts that higher factor concentrations will drive non-physiological protein interactions or, in the case of transcription factors, DNA binding. Therefore, it is often asserted that overexpression of a transcription factor will not accurately reflect the function of that factor at physiologic levels of expression or, at a minimum, that there is no basis to assume that it does. As logical as this assertion may seem, there is very little experimental evidence to support or refute it, possibly because genome-wide factor binding and transcriptional activation have only recently been possible to assess. Yet, understanding the functional consequences of transcription factor overexpression is very important in cancer cell biology where gene amplifications, such as *N-MYC* amplification in neuroblastomas, promote tumor progression.

Previously we reported the genome-wide binding of endogenous MyoD in mouse muscle cells and compared that to exogenously expressed MyoD in mouse embryonic fibroblasts (MEFs) transduced with a MyoD expressing retrovirus [1]. The transduced MEFs had levels of MyoD protein very similar to the endogenous MyoD and showed a very similar binding profile. Here we compare the genome-wide binding of endogenous MyoD in mouse skeletal muscle cells with highly overexpressed MyoD in MEFs to determine whether overexpression qualitatively alters the binding profile. We find that the overexpressed MyoD binds to the same sites as endogenous MyoD and does not demonstrate binding to novel regions or motifs. Our study shows that overexpression of MyoD accurately identifies sites bound by endogenous MyoD, suggesting an intrinsic biological robustness for varying levels of transcription factor in a cell.

## Methods

### ChIP-seq

ChIP was performed as previously described [1,2]. ChIP samples were prepared for sequencing per the Illumina Sample Preparation protocol with two modifications: (1) DNA fragments of 150–300 bp were selected at the gel-selection step; (2) 21 cycles of PCR were performed at the amplification step instead of 18. For the control samples, untransduced MEFs derived from *Myod*^*−/−*^*/Myf5*^*−/−*^ mice were ChIPed with MYOD antibody, and mouse myotubes were ChIPed with preimmune serum.

### MyoD lentivirus

cDNA for *Myod* was cloned into the GFP locus of the pRRL.SIN.cPPT.PGK-GFP.WPRE lentiviral backbone (Addgene), with expression thus driven by the *Pgk* promoter. Replication-incompetent lentiviral particles were packaged in 293T cells by the Fred Hutchinson Cancer Research Center Lentivirus Core Facility. MEFs were transduced in DMEM containing polybrene at 8 μg /ml. After 24 h, media were replaced; cells were switched to differentiation media (1% heat inactivated horse serum, 10 μg/ml insulin, 10 μg/ml transferrin) 48 h after infection and harvested 36–40 h later. ChIP and Western blot were performed with a previously characterized MYOD antibody [3]. Western blot bands were quantified with ImageJ.

### ChIP-seq peak calling and significance inference

Sequences were extracted using the GApipeline software. Reads mapping to the X and Y chromosomes were excluded from our analysis. Reads were aligned using MAQ to the mouse genome (mm9). Duplicate sequences were discarded to minimize effects of PCR amplification. Each read was extended in the sequencing orientation to a total of 200 bases to infer the coverage at each genomic position. Peak calling was performed by an in-house developed R package that models background reads by a negative binomial distribution conditioned on GC content as previously described [1,2,4]. The control ChIP-seq sample was used to eliminate statistically significant peaks likely due to artifact.

### Motif analysis

We used a discriminative *de-novo* motif discovery tool described previously [2] to find motifs that distinguish foreground and background sequence data sets. To find motifs enriched under ChIP-seq peaks, we selected background sequences using random genomic regions sampled with similar GC content and distance to TSS. We infer a positional weight matrix (PWM) model from an output motif using an iterative expectation-maximization (EM) refinement process, which is similar to MEME [5].

### ChIP-seq sample comparison

The scatter plot of the MyoD peak heights in the two samples (endogenous MyoD and lenti-MyoD) indicated a strong correlation (Pearson correlation 0.52 with asinh transformation). Nevertheless, due to the different origins of samples, the overall variation between the two was still far greater than their respective technical replicates (data not shown). Therefore, it was challenging to apply an appropriate statistical null model to capture the stochastic variation between the two systems and to identify exactly the set of peaks that are identical or different between the samples. Therefore, we chose a non-parametric approach by comparing the overlap of peaks at a spectrum of different rank cutoffs in order to outline the global landscape of peak similarity. Cross cell-type comparison was performed similarly as previously described [4]. We ranked all peaks by their p-values and group ranks into bins of 5,000 (i.e., the top 5K peaks, then the top 10K peaks, etc.). Then we computed the fraction of the top x peaks in one sample that overlap with the top y peaks in another sample, where x and y vary from 5K to 110K, and y is equal to or greater than x. To compare the coverage at E-boxes in endogenous and lenti peaks, and to quantify the distribution of peak height ratios between the two samples, we adjusted for the different numbers of total reads by sub-sampling equal numbers of endogenous and lenti reads and recomputed the coverage and peak height at these sites.

### Ethical approval

This study did not directly use vertebrate animals or human subjects and did not require ethical approval.

## Results

### Comparison of different MyoD expression levels in the conversion of fibroblasts to skeletal muscle

**Figure 1:**
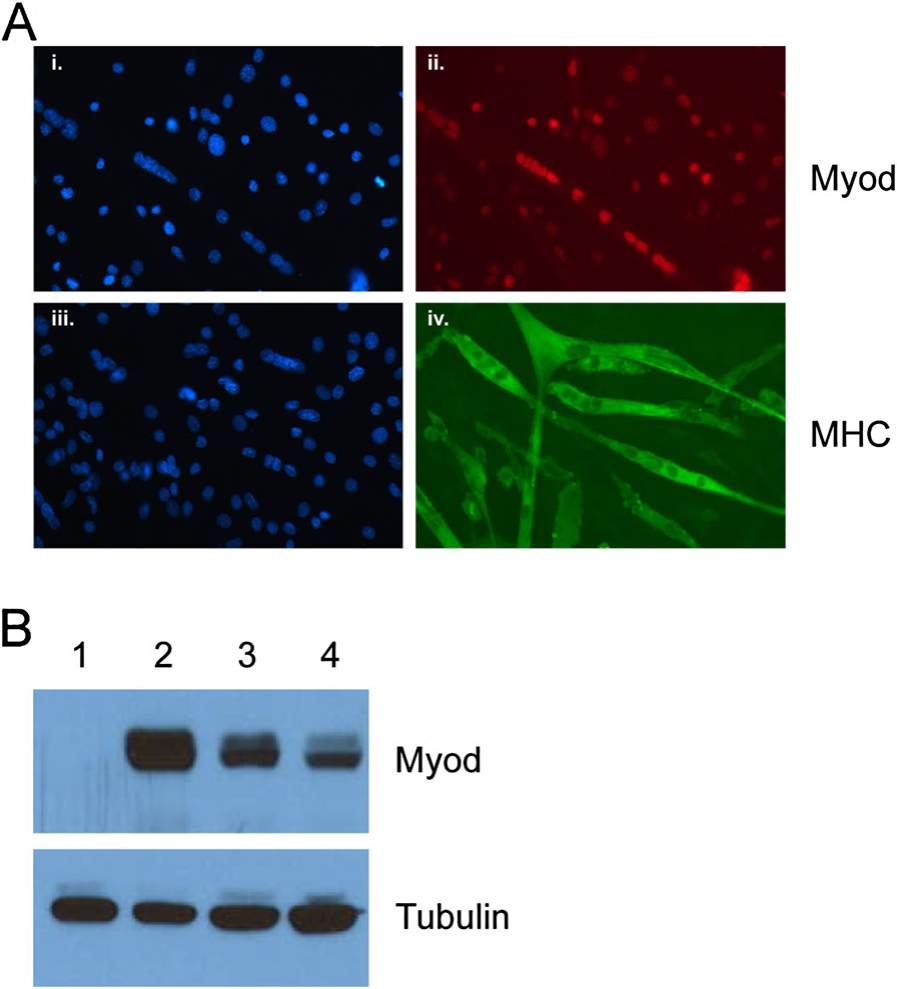
**MEFs transduced with MyoD lentivirus.** (**A**) MEFs transduced with MyoD lentivirus demonstrate nearly complete conversion into myotubes 72 h after infection (*red*: MYOD antibody; *green*: myosin heavy chain antibody; *blue*: DAPI). (**B**) Western blot demonstrates higher MyoD expression in MEFs transduced with MyoD lentivirus (*2*) compared to control MEFs (*1*), C2C12 myoblasts (*3*), and C2C12 myotubes (*4*). Tubulin blot demonstrates equivalent loading.

Mouse embryonic fibroblasts (MEFs) can be converted to skeletal muscle by the forced expression of MyoD. To determine whether overexpression of MyoD can reliably identify biologically relevant binding sites, we compared the binding profile of the endogenous MyoD in mouse muscle cells to the MyoD binding profile in MEFs with exogenously overexpressed MyoD. Transduction of MEFs with a lentivirus expressing MyoD from the *Pgk* promoter (lenti-MyoD) induced differentiation to skeletal muscle as determined by fusion and expression of myosin heavy chain (Figure 1A). Western analysis demonstrated that lenti-MyoD cells had approximately four-fold higher levels of MyoD protein than the endogenous MyoD of C2C12 myotubes (Figure 1B).

### Overexpressed MyoD binds the same sites as endogenous MyoD

To determine whether overexpressed MyoD can accurately identify sites bound by endogenous MyoD, we compared a ChIP-seq data set obtained from MEFs transduced with lenti-MyoD [4] to our previous ChIP-seq data from endogenous MyoD in mouse myotubes [1]. The lenti-MyoD data set had 17.5 million mapped unique reads, and we combined endogenous MyoD ChIP-seq data from mouse C2C12 myotubes (6.5 million reads) and primary differentiated cultured mouse muscle cells (8.5 million reads) to achieve a comparable total 15 million reads for endogenous MyoD because our prior analysis [1] demonstrated a high concordance of peak locations between these two samples. These reads were processed and peaks were identified as described in Methods and Additional file 1: Figure S1A and B. The control ChIP-seq samples (~18 million pooled reads from pre-immune ChIP in muscle cells, MyoD antisera ChIP in MEFs that do not express MyoD, and beads alone) contained a small number of high peaks (Additional file 1: Figure S1B), which were found at similar locations in all three control ChIP samples (data not shown) and remain of unknown etiology. These non-MyoD peaks were subtracted from the MyoD ChIP-seq data sets.

ChIP-seq for the endogenous MyoD identified ~37,000 peaks at a *p*-value (see Methods) of 10^-10^ (read cutoff ~20), ~67,000 peaks at a *p*-value of 10^-5^ (read cutoff ~11), and ~117,000 at a *p*-value of 10^-3^ (read cutoff ~8). A similar range of peaks was identified by the overexpressed MyoD but at slightly higher *p*-value thresholds: ~35,000 (p~10^-20^, cutoff ~50), ~68,000 (p~10^-10^, cutoff ~26), and ~122,000 (p~10^-5^, cutoff ~14). At a given *p*-value, the overexpressed MyoD had approximately twice the number of peaks compared to the endogenous MyoD. This could either represent higher occupancy of the same sites bound by the endogenous MyoD or a large number of off-target sites bound by the overexpressed MyoD and not bound by the endogenous MyoD.

**Figure 2:**
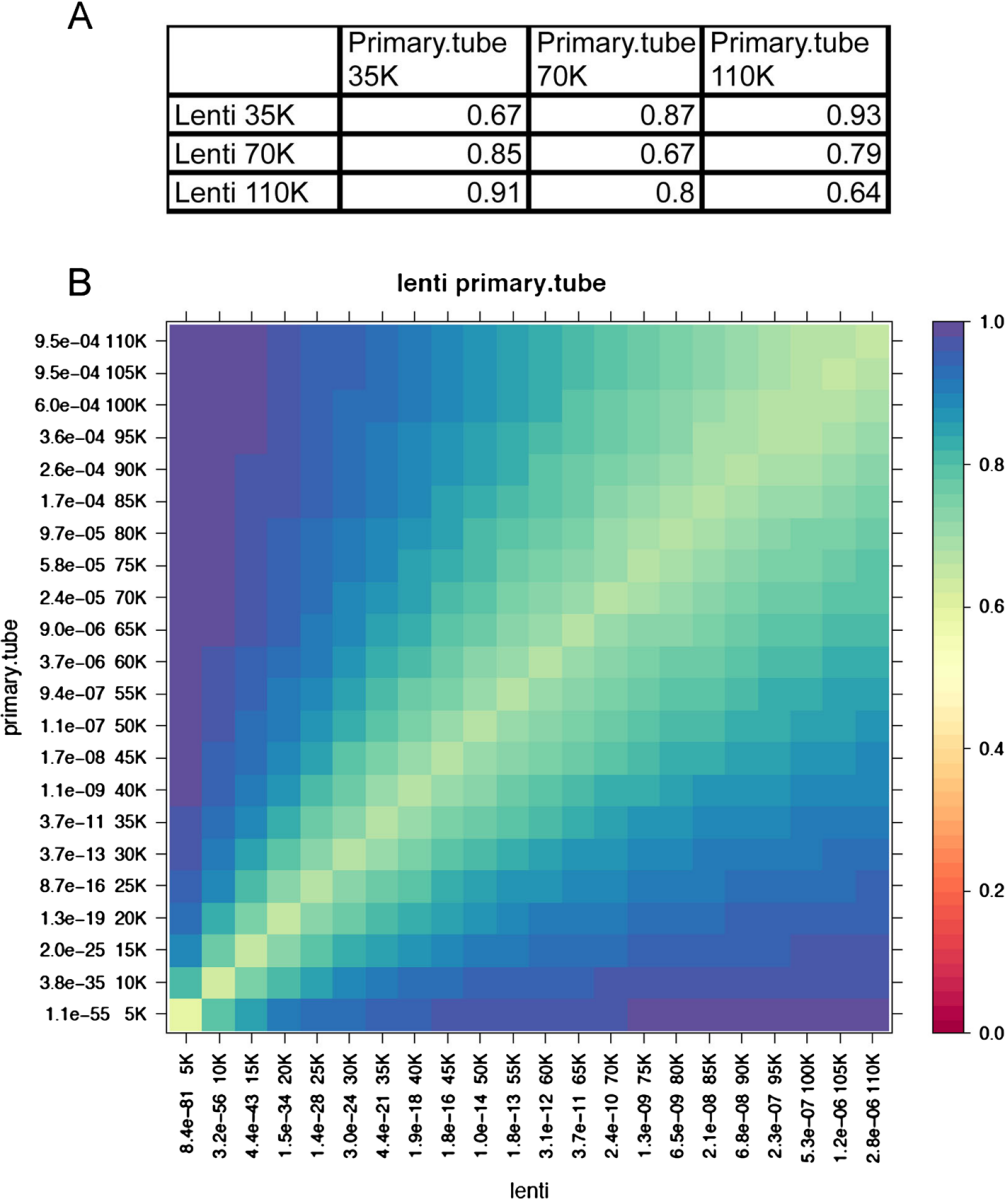
**The MyoD binding regions are largely shared between overexpressed MyoD in MEFs and endogenous MyoD.** (**A**) Overlap of lenti-MyoD peaks with endogenous MyoD peaks. To assess the concordance between the two samples, we selected the top 5K to 110K peaks in each sample based on *p*-value. We calculated the number of overlapping peaks for the top peak sets at various rank cutoffs in both samples and divided this number by the size of the smaller peak set. Specifically, for a cell corresponding to the top x peaks in sample 1 compared to the top y peaks in sample 2, the fraction is computed as the number of overlapping peaks divided by the smaller value of x or y. (**B**) The overlapping fractions were calculated as in A and are plotted with color-coding as specified in the figure. For example, in the cell at the row labeled 5K and column labeled 10K, we plotted the fraction of the top 5K peaks in lenti-MyoD that overlap with the top 10K peaks in primary + C2C12 myotubes.

To accurately compare the similarity, or overlap, of MyoD binding sites in the different samples, we used a non-parametric approach that compared the overlap of peak locations based on the rank order of the peaks in each sample (see Methods). Comparing the top 35,000 peaks bound by endogenous MyoD and lenti-MyoD, there was a 67% overlap, and the overlap was similar comparing the top 70,000 or 110,000 peaks for each (Figure 2A). The lack of a complete overlap at each cutoff was largely due to the rank-order of the peaks (based on p-value) rather than distinct binding regions, because 87% of the top 35,000 lenti-MyoD peaks were represented in the top 70,000 endogenous-MyoD peaks and 93% in the top 110,000 endogenous-MyoD peaks. Similarly, 85% and 91% of the top 35,000 endogenous-MyoD peaks were present in the top 70,000 and 110,000 lenti-MyoD peaks, respectively. A more detailed representation of this data is shown in Figure 2B. Therefore, although there were some differences in the rank order of the peaks, the locations were almost the same with greater than 90% concordance.

### Overexpressed MyoD binds the same motifs as endogenous MyoD

Since MyoD binds as a heterodimer with an E-protein to an E-box containing a CANNTG core sequence, with preference for GC or CC as the internal nucleotides [1,4], we next determined whether overexpression of MyoD resulted in binding to a distinct set of low affinity sites or sites that might reflect homodimers or other protein complexes. We used two different approaches to determine the binding site preferences for endogenous and overexpressed MyoD.

**Figure 3:**
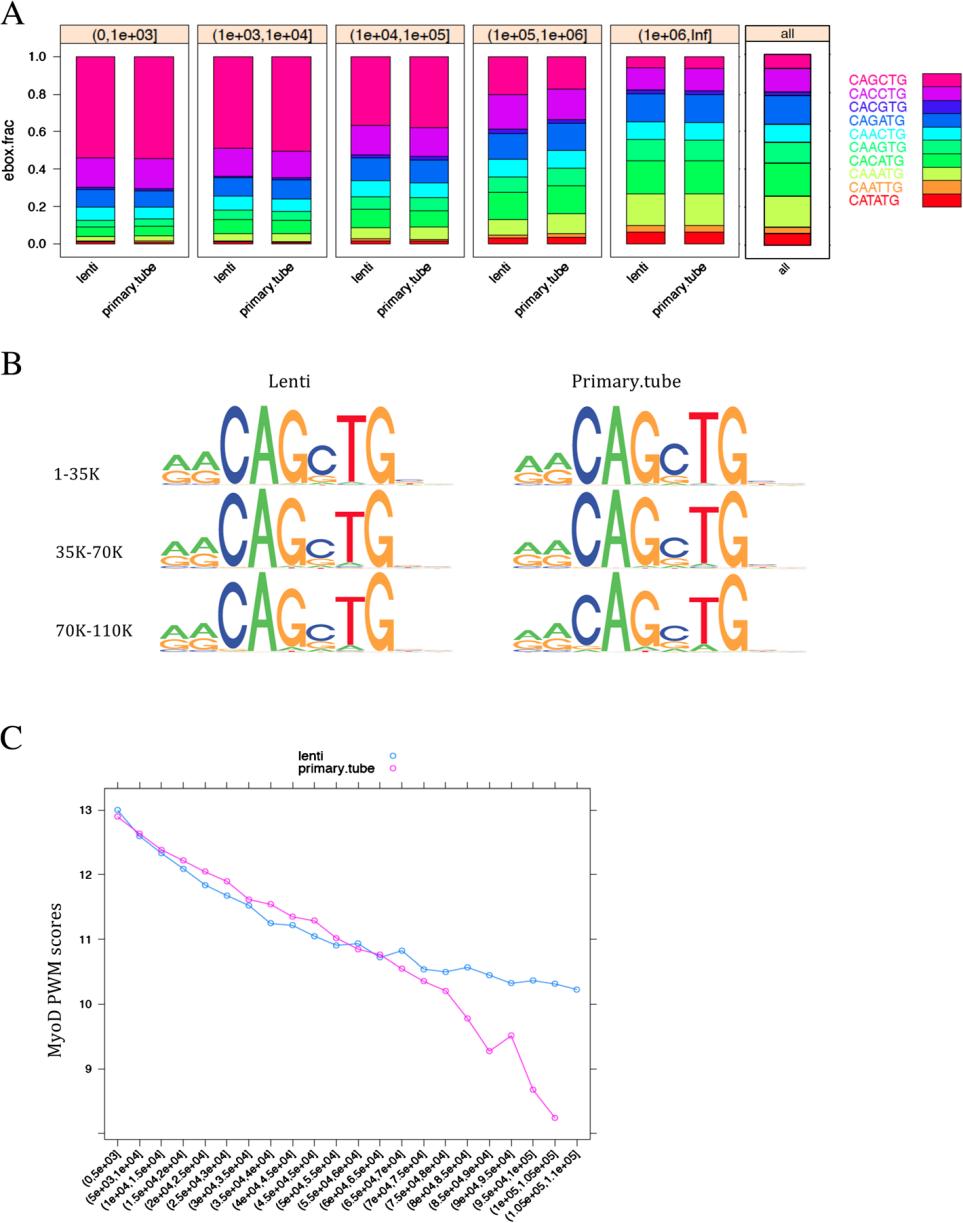
**MyoD has a similar E-box preference for both endogenous and overexpressed MyoD.** (**A**) Overexpressed MyoD (lenti) and endogenous MyoD (primary.tube) have similar E-box distributions. We collected all genomic E-boxes (excluding sex chromosomes and those present in the peaks of the control samples) and ranked them based on *p*-values for the read coverage at the E-boxes. We partitioned them into bins of top 1K, top 1001 to 10K, etc., until all E-boxes are included. Within each bin, we calculated the percentage of each type of E-box variant, and plotted the distribution. The background E-box distribution over the entire genome is also included as a reference. (**B**) MYOD binding sites for overexpressed MyoD (lenti) and endogenous MyoD (primary.tube) share the same sequence preference. We used motif discovery to identify the E-box motif under MyoD bound peaks for the top 35K, the top 35K+1 to 70K peaks, and the top 70K+1 to 110K peaks. The E-box sequence preferences are nearly identical, including within the flanking regions. Motifs in lower ranking peaks tend to have slightly more sequence degeneracy. (**C**) MyoD E-box average PWM score compared to peak rank. The MyoD PWM is derived from our previous study [4]. Weak peaks tend to have weaker motifs, but the degradation is more gradual for overexpressed MyoD peaks (lenti) beyond the top 67K, suggesting that a subset of noisy low peaks in the endogenous MyoD (primary.tube) is elevated to reasonably strong peaks distinguished from the background. X-axis: the peak rank bins. Y-axis: the average PWM scores for all peaks within the rank bin.

First, we ranked all of the approximately 15 million E-boxes in the mouse genome based on their ChIP-seq coverage (see Methods for details of the statistical model) and then binned them by rank as the top 1,000, 1,001–10,000, 10,001-100,000, etc. For both endogenous and lenti-MyoD, the CAGCTG and CACCTG E-boxes were enriched for MyoD binding (Figure 3A) in similar proportions within each bin. Therefore, overexpressed MyoD binds a similar distribution of E-box sequences as the endogenous MyoD.

For the second approach to determine whether overexpression of MyoD resulted in binding to a different set of E-boxes, we used a motif discovery algorithm to identify preferred E-box sequences associated with the top 35,000 ranked peaks (ranked on *p*-value), the 35,001-70,000 peaks, and the 70,001-110,000 peaks for both endogenous MyoD and overexpressed MyoD (Figure 3B). The identified E-box motifs, and flanking preferences were nearly identical for endogenous MyoD and overexpressed MyoD at different rankings, although the lower ranked peaks had a slightly more degenerate sequence compared to the higher ranked peaks in both groups. Plotting the average position weight matrix (PWM) score for the highest PWM E-box under each peak against the rank of the peaks demonstrates that the average PWM for the low ranked peaks (rank > 85,000) falls off more rapidly for the endogenous MyoD compared to the lenti-MyoD (Figure 3C). This likely reflects the difficulty of accurately discriminating weak MyoD binding sites from background reads for the endogenous MyoD, whereas the lenti-MyoD maintains a higher average E-box PWM score, suggesting that the overexpression of MyoD enhances the ability to discriminate weaker MyoD binding sites from background. Taken together, the data indicate that overexpressed MyoD in MEFs binds to a nearly identical set of sites and E-boxes as the endogenous MyoD in mouse muscle cells without a substantial number of off-target sites.

**Figure 4:**
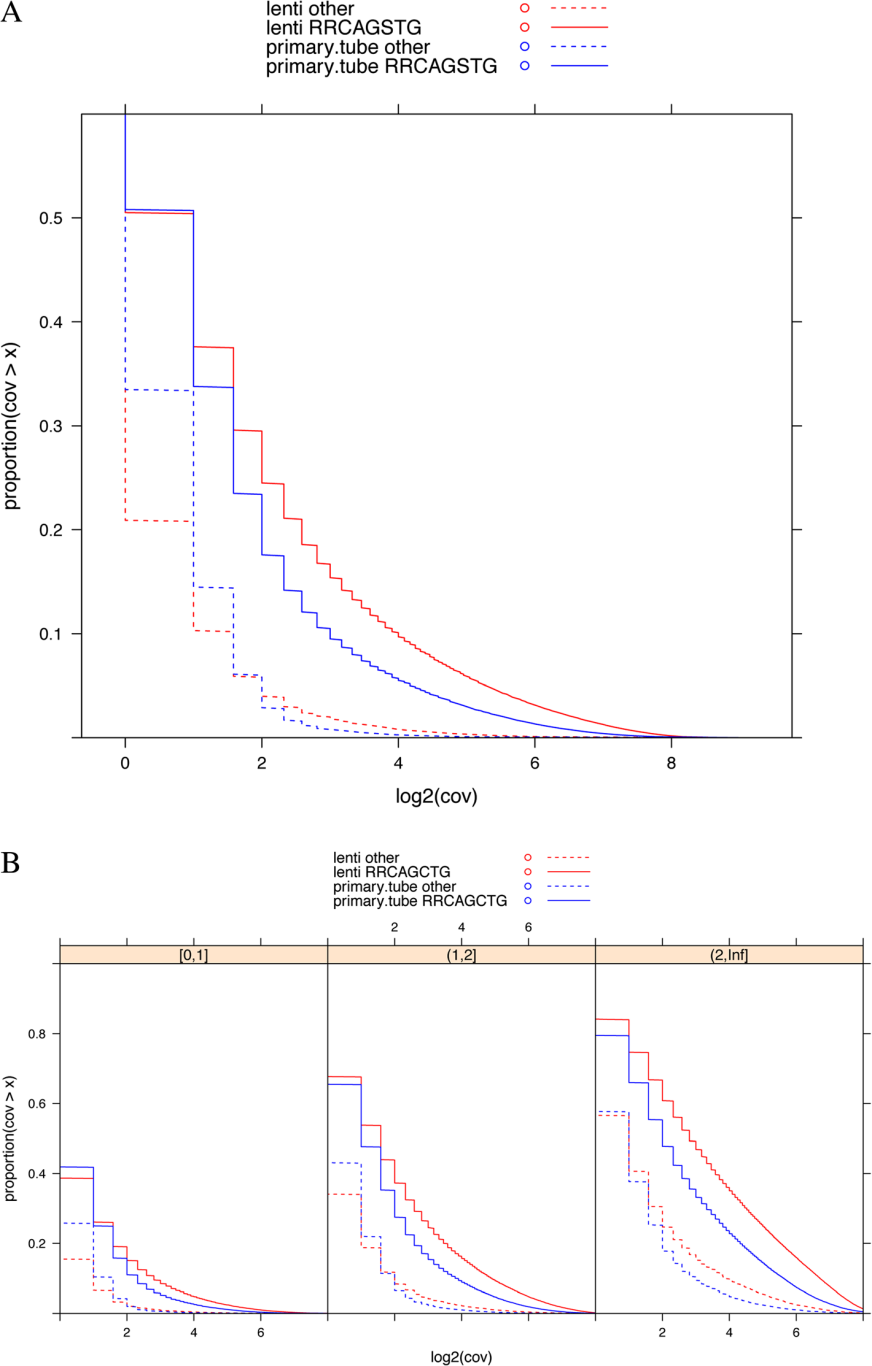
**MyoD binds a subset of accessible E-boxes.** (**A**) Coverage distribution over consensus MyoD binding sites RRCAGSTG. X-axis: log2 transformed coverage. Y-axis: the proportion of sites with coverage greater than the given value x. To make read coverage more comparable, we sub-sampled the same number of reads in each group. The distributions of coverage at RRCAGSTG sites in both samples are shown in *solid lines*. For comparison, E-boxes other than RRCAGSTG sites are shown in *dashed lines*. Only the E-boxes that are uniquely mapped within a ±200-bp window are included. (**B**) Coverage distribution over E-boxes similar to panel **A** but divided into E-boxes showing relatively low accessibility (0,1], moderate accessibility (1,2], or relatively high accessibility (2,Inf], as previously determined by PvuII accessibility [4].

To determine whether overexpressed MyoD was bound to all of the E-boxes that match the consensus site, we graphed the number of reads over all the RRCAGSTG sites in the mappable genome (Figure 4A), sub-sampling the same number of reads for both the overexpressed and endogenous MyoD ChIP-seq data sets. Despite overexpression in the lenti-MyoD samples, the majority of these high PWM E-boxes had one read or less, similar to the endogenous-MyoD samples, and for both samples fewer than 25% of these high PWM E-boxes had more than four reads. Therefore, for both the endogenous and overexpressed MyoD, a minor subset of high PWM E-boxes was occupied by MyoD.

Previously, we had measured E-box accessibility in fibroblasts prior to the expression of MyoD by exposing isolated nuclei to the restriction enzyme PvuII, which cleaves at CAGCTG E-boxes [4]. Using these data, we assigned each CAGCTG E-box to one of three groups: relatively inaccessible, moderately accessible, or highly accessible. About 40% of all highly accessible CAGCTG E-boxes had more than four reads in the endogenous MyoD samples, and slightly over 50% had four or more reads in the overexpressed MyoD samples; this compares to approximately 15% for both samples over the relatively inaccessible E-boxes (Figure 4B). However, only about one-third of the highly accessible group had read coverage above the average cutoff for the top ~70,000 peaks (11 reads for endogenous MyoD and 26 reads for overexpressed MyoD). Therefore, E-box accessibility in the chromatin was a major determinant of MyoD binding, but a large fraction of relatively highly accessible E-boxes with a strong PWM remained unbound by MyoD, indicating that only a subset of accessible E-boxes with a good PWM showed substantial MyoD binding even when MyoD was overexpressed. In this regard, our prior study [4] showed that several sequence motifs were enriched in the region of bound accessible sites (additional E-boxes, higher PWM E-boxes, and a motif similar to a MEIS binding site), indicating that several factors might operate to enhance or stabilize MyoD binding at subsets of accessible sites.

### Co-factor motifs and MyoD binding

Although there was a very high concordance of binding sites for endogenous MyoD in mouse muscle cells and overexpressed MyoD in MEFs, there was some difference in peak rank, as evidenced by a 67% overlap of the top 35,000 peaks in each set with most of the additional 33% of peaks present in the other set at lower rank. Since the E-box motifs were similar in both sets and did not apparently account for rank differences, we examined the top 30,000 peaks in each set for co-factor motifs using a *de novo* motif search strategy (see Methods). The peaks in the mouse muscle cells were enriched in E-box motifs (>7-fold) and had modest enrichment for several other motifs: MEIS (1.6-fold), RUNX (1.3-fold), and AP1 (2.2-fold). Similarly, the peaks in the MEFs with overexpressed MyoD were enriched for E-box motifs (>6-fold), MEIS (1.6-fold), and RUNX (1.4-fold) relative to the background sequence (Additional file 1: Figure S2A). It remains possible that some of the rank differences reflect the relative abundance of co-factors in the different cell backgrounds, but there is not a strong association of a specific factor motif with the MyoD binding sites.

Although there was about 90% concordance between endogenous and overexpressed MyoD peaks, albeit with some difference in ranking position, approximately 5-7% of the highly ranked peaks in each set was not represented in the top 110,000 peaks in the other set. Motif analysis of the sequences under the endogenous-only peaks (i.e., ranked in the top 30,000 endogenous peaks but not in the top 110,000 lenti-MyoD peaks) compared to peaks only present in the lenti-MyoD (i.e., ranked in the top 30,000 lenti-MyoD peaks but not in the top 110,000 endogenous MyoD peaks) showed an enrichment of a variant of the RUNX motif (2-fold), a PITX-like motif (4-fold), and a CGNCAG motif (2.7-fold). A similar motif analysis comparing the endogenous-only peaks to shared peaks also revealed the RUNX and PITX-like motifs, albeit at a slightly lower fold enrichment. The comparable analysis to identify motifs in the lenti-only peaks revealed a slight enrichment for E-boxes with non-preferred core sequences (Additional file 1: Figure S2B), indicating that a small number of the lenti-only peaks might represent binding to lower affinity sites, possibly driven by the higher amount of MyoD. Therefore, while there might be some contribution of co-factors expressed in the mouse muscle cells that accounts for the small number of endogenous-only peaks, the motif analysis does not identify more than a modest enrichment of the motif for any specific factor, consistent with the finding that the MyoD binding sites in both cells types show over 90% concordance.

## Discussion

We conclude that overexpression of MyoD can accurately identify endogenous MyoD binding sites. This is true despite the fact that the binding pattern of the endogenous MyoD was determined in skeletal muscle cells (primary myotubes and differentiated C2C12 cells), whereas the binding sites of the overexpressed MyoD were determined in MEFs. The concordance of binding sites in these two cell types might reflect the ability of MyoD to convert MEFs to skeletal muscle. In this process, MyoD activates the expression of many co-factors that cooperate in a feed-forward circuit with MyoD to orchestrate gene expression [7-9]. Since MyoD can activate its own co-factors, the initial differences in co-factor expression between muscle cells and MEFs might not alter the ultimate binding pattern of MyoD.

In differentiating muscle cells, MyoD binds DNA as a heterodimer with an E-protein. However, *in vitro* binding studies demonstrate that MyoD can form homodimers and bind E-boxes. Therefore, we had anticipated that overexpressed MyoD might bind as a homodimer because of limiting amounts of E-proteins. Surprisingly, we do not think there is any evidence for homodimer binding. The E-box motif analysis of the endogenous MyoD has asymmetric flanking sequences: RRCAGSTG. In a recent study we have shown that NeuroD2 binds an E-box with similar flanking preferences on one side: RRCAGMTGG [4]. Because both MyoD and NeuroD2 form heterodimers with the same E-proteins, we assume that the flanking RR is determined by the common E-protein partner, and initial binding studies support this conclusion (AP Fong, unpublished data). Since these flanking preferences are maintained at the E-boxes when MyoD is overexpressed, it suggests that the E-protein determined sequence preference is maintained and that MyoD is binding as a heterodimer even when overexpressed. It is possible that the requirement for heterodimer binding prevents off-target DNA binding by the overexpressed MyoD since the amount of the E-protein dimerization partner would be limiting.

The fact that overexpression of MyoD improved the foreground-to-background signal and permitted site determination at higher p-value stringency suggests that many of the MyoD binding sites might not be occupied 100% of the time at endogenous levels of MyoD, although this remains speculative since other unknown variables might have affected the ChIP efficiencies or foreground/background read ratios in the different experiments. With these caveats in mind, if the majority of sites are not saturated by physiological levels of MyoD (i.e., not bound by MyoD 100% of the time) then these would present a large “sink” for the overexpressed MyoD protein, which might further limit ectopic binding. In this regard, it is interesting to note that re-analysis of published c-Myc binding ChIP-seq data [6] under low and high serum conditions that result in an approximately five-fold change in *c-Myc* mRNA also shows enhanced binding of weakly bound sites with increased c-Myc levels (ZY and SJT, unpublished data). Furthermore, while this manuscript was under review, Lin et al. [10] demonstrated that increased amounts of c-Myc protein resulted in greater saturation of weakly bound c-Myc sites near promoters and this was associated with increased gene transcription. Additional studies will be required to determine whether increased MyoD binding at physiologically unsaturated sites has a similar function in enhancing gene transcription. Together these findings suggest that transcription factor overexpression, e.g., induced by gene amplification or other mechanisms in cancers, might have major biological consequences as a result of increased binding at physiologically bound sites.

## Conclusions

Our comparison of genome-wide binding of endogenous MyoD with overexpressed MyoD demonstrated that overexpressed MyoD binds to the same sites as endogenous MyoD and does not demonstrate binding to novel regions or motifs. The samples with overexpressed MyoD showed better foreground-to-background signal and permitted site determination at higher statistical significance, suggesting that increased amounts of transcription factor increased the binding at physiologically bound sites. Overall, our study shows that overexpression of MyoD accurately identifies sites bound by endogenous MyoD and demonstrates an intrinsic biological robustness for varying levels of transcription factor in a cell.

### Accession numbers

ChIP-seq data have been deposited in Gene Expression Omnibus (GEO) under accession number GSE34906 (lenti-overepressed MyoD) and in DDBJ Sequence Read Archive (DRA) accession number SRP001761 (endogenous MyoD).

## Abbreviations

MEFs: Mouse embryonic fibroblasts; lenti-MyoD: MEFs transduced with MyoD lentivirus; PWM: Position weight matrix; EM: Expectation-maximization.

## Competing interests

The authors declare that they have no competing interests.

## Authors’ contributions

ZY performed the computational analysis; APF and YC performed the experiments; WLR and RCG provided oversight for the computational analysis; SJT provided oversight for the biological experiments; all authors participated in the experimental design and interpretation. All authors read and approved the final manuscript.

## Supplementary Material

Additional file 1: Figure S1 (**A**) The percentage of genome covered at a given *p*-value significance. X-axis corresponds to the negative logarithm of the *p*-value significance level, and Y-axis is the fraction of genome covered, both in log10 scale. The pink curve (*ref*) corresponds to the estimated percentage of genome covered at the given *p*-value cutoff based on the null hypothesis in each sample, and the blue curve (*obs*) is the observed percentage of genome covered at the given *p*-value cutoff. FDR is defined as the ratio of observed vs. background genome covered at a given *p*-value. The three panels correspond to overexpressed MyoD (lenti), endogenous MyoD (primary.tube), and the control samples (control). (**B**) Pairwise comparison of control samples. We have three types of control: pooled reads from pre-immune ChIP in muscle cells (Tube preimmune), MyoD antisera ChIP in MEFs that do not express MyoD (MEF control), and beads alone (MEF bead). Reads from all control lanes are pooled to infer peaks at very low significance (*p*-value 10^-3^), and we calculate the maximum coverage for each sample at these peaks. The pairwise comparison of coverage of each sample in square root transformation is shown. **Figure S2:** Motif enrichment analysis for regions under overexpressed MyoD peaks and endogenous MyoD peaks. (**A**) Motifs enriched under all overexpressed MyoD peaks (lenti) or all endogenous MyoD peaks (primary.tube). (**B**) Motifs specific to endogenous or overexpressed MyoD peaks. Primary-Lenti: Motifs enriched in peaks present only in endogenous MyoD compared to peaks present only in overexpressed MyoD. Primary-Shared: Motifs enriched in endogenous-only peaks compared to peaks present in both groups, i.e., shared peaks. Lenti-Shared: Motifs enriched in peaks only in overexpressed MyoD compared to shared peaks. *Consensus*, consensus sequence for the motif; *Anno*, annotated factor for motif consensus; *scores*, the regression z-values representing the discriminative power of the motif for separating the foreground and background where positive values indicate enriched motifs and negative values indicate depleted motifs; *ratio*, the enrichment (or depletion) ratio of the motifs in the foreground relative to the background; *fg.frac*, the percentage of the foreground sequences containing the motif; *bg.frac*, the percentage of the background sequences containing the motif; *logo*, the PWM logo. Click here for file
