# Supplementary material for: Comparison of endogenous and overexpressed MyoD shows enhanced binding of physiologically bound sites
Source: Skelet Muscle. 2013 Apr 8;3:8. doi: 10.1186/2044-5040-3-8 (PMC3666993; doi:10.1186/2044-5040-3-8)
Supplement: Additional file 1: Figure S1 — (A) The percentage of genome covered at a given p-value significance. X-axis corresponds to the negative logarithm of the p-value significance level, and Y-axis is the fraction of genome covered, both in log10 scale. The pink curve (ref) corresponds to the estimated percentage of genome covered at the given p-value cutoff based on the null hypothesis in each sample, and the blue curve (obs) is the observed percentage of genome covered at the given p-value cutoff. FDR is defined as the ratio of observed vs. background genome covered at a given p-value. The three panels correspond to overexpressed MyoD (lenti), endogenous MyoD (primary.tube), and the control samples (control). (B) Pairwise comparison of control samples. We have three types of control: pooled reads from pre-immune ChIP in muscle cells (Tube preimmune), MyoD antisera ChIP in MEFs that do not express MyoD (MEF control), and beads alone (MEF bead). Reads from all control lanes are pooled to infer peaks at very low significance (p-value 10-3), and we calculate the maximum coverage for each sample at these peaks. The pairwise comparison of coverage of each sample in square root transformation is shown. Figure S2: Motif enrichment analysis for regions under overexpressed MyoD peaks and endogenous MyoD peaks. (A) Motifs enriched under all overexpressed MyoD peaks (lenti) or all endogenous MyoD peaks (primary.tube). (B) Motifs specific to endogenous or overexpressed MyoD peaks. Primary-Lenti: Motifs enriched in peaks present only in endogenous MyoD compared to peaks present only in overexpressed MyoD. Primary-Shared: Motifs enriched in endogenous-only peaks compared to peaks present in both groups, i.e., shared peaks. Lenti-Shared: Motifs enriched in peaks only in overexpressed MyoD compared to shared peaks. Consensus, consensus sequence for the motif; Anno, annotated factor for motif consensus; scores, the regression z-values representing the discriminative power of the motif for separating the foreground and [file 2044-5040-3-8-S1.doc]

Supplemental Figure 1A.


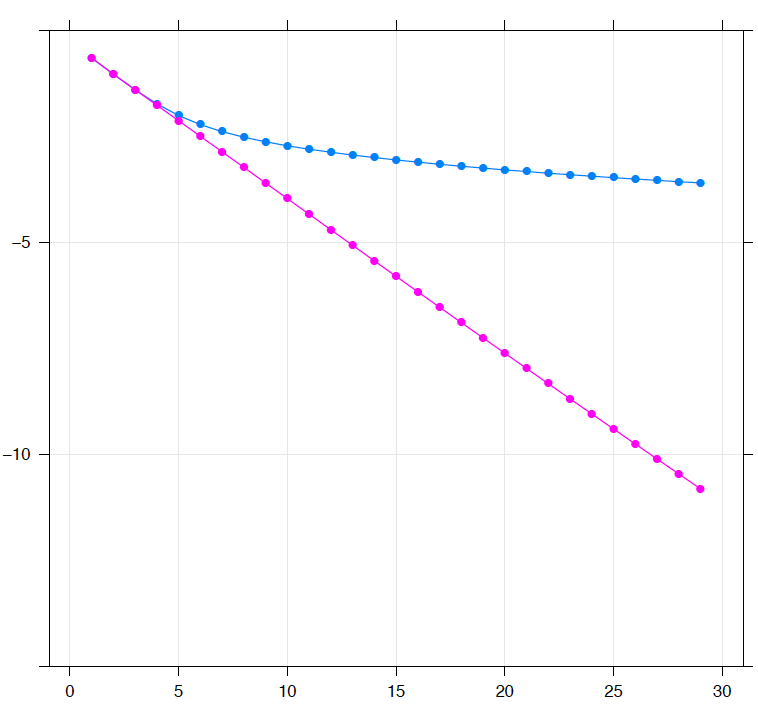

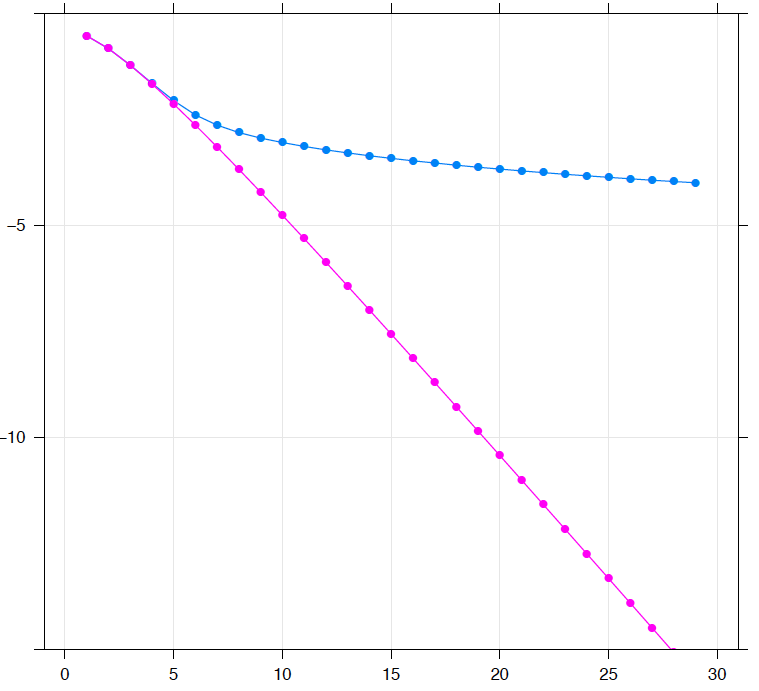

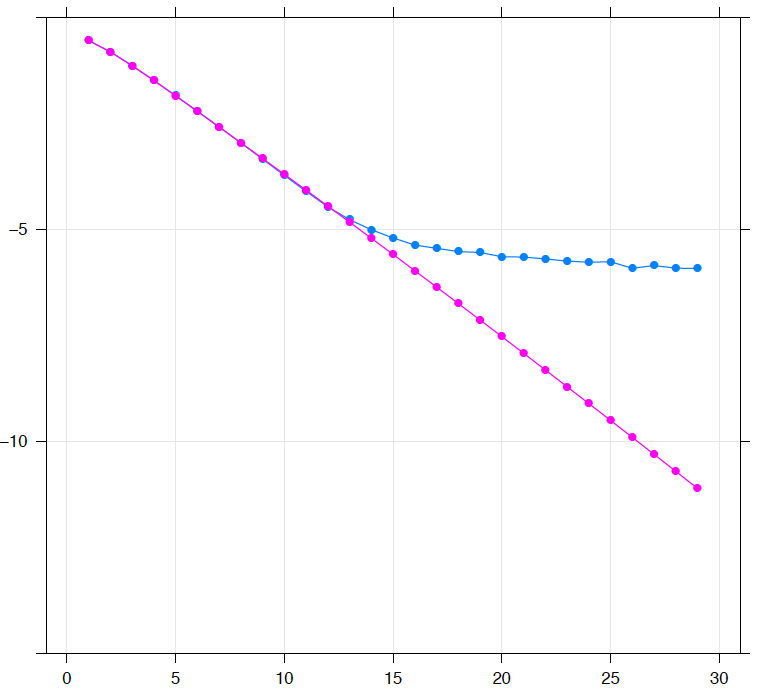


Primary.tubee

Lenti

Control

Supplemental Figure 1B.

**Supplemental Figure 1.** (A). The percentage of genome covered at a given p-value significance. X-axis corresponds to the negative logarithm of the pvalue significance level, and Y-axis is the fraction of genome covered, both in log10 scale. The pink curve (ref) corresponds to the estimated percentage of genome covered at the given pvalue cutoff based on the null hypothesis in each sample, and the blue curve (obs) is the observed percentage of genome covered at the given p-value cutoff. FDR is defined as the ratio of observed vs. background genome covered at a given pvalue. The three panels correspond to over-expressed MyoD (lenti), endogenous MyoD (primary.tube), and the control samples (control). (B). Pairwise comparison of control samples. We have three types of control: pooled reads from pre-immune ChIP in muscle cells (Tube preimmune), MyoD antisera ChIP in MEFs that do not express MyoD (MEF control), and beads alone (MEF bead). Reads from all control lanes are pooled to infer peaks at very low significance (pvalue 10^-3^), and we calculate the maximum coverage for each sample at these peaks. The pairwise comparison of coverage of each sample in square root transformation is shown.

Supplemental Figure 2A.


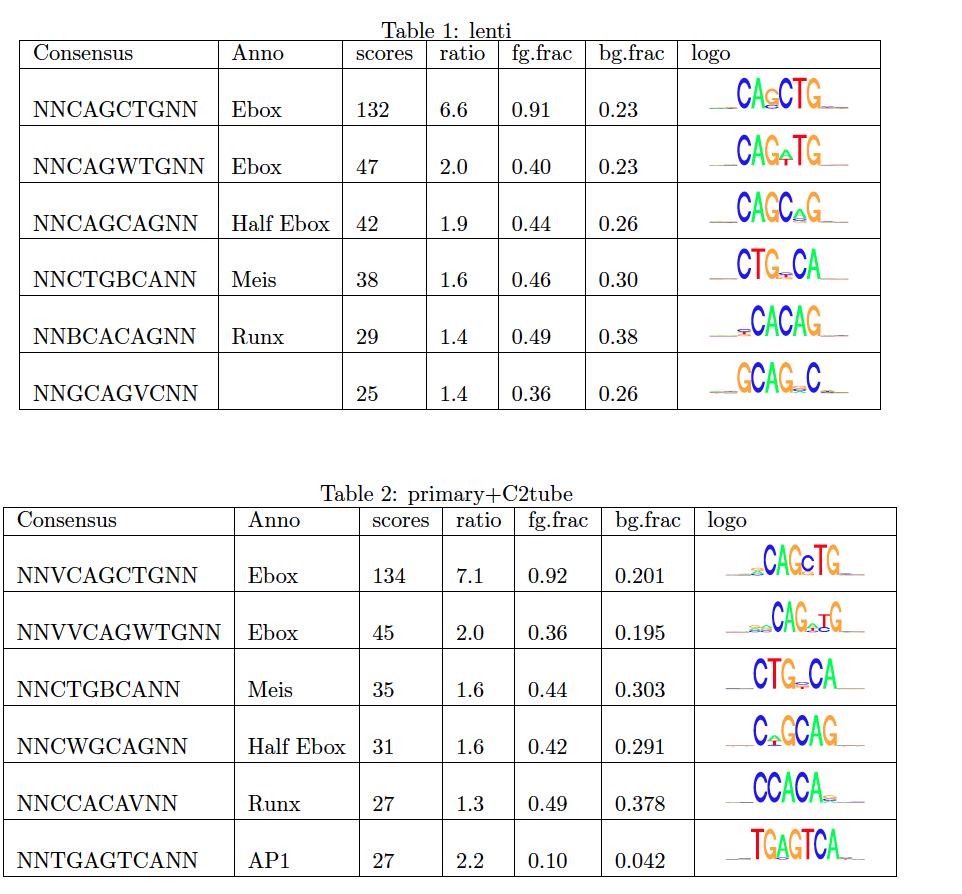


Enriched Motifs in Primary.tube MyoD peaks against background


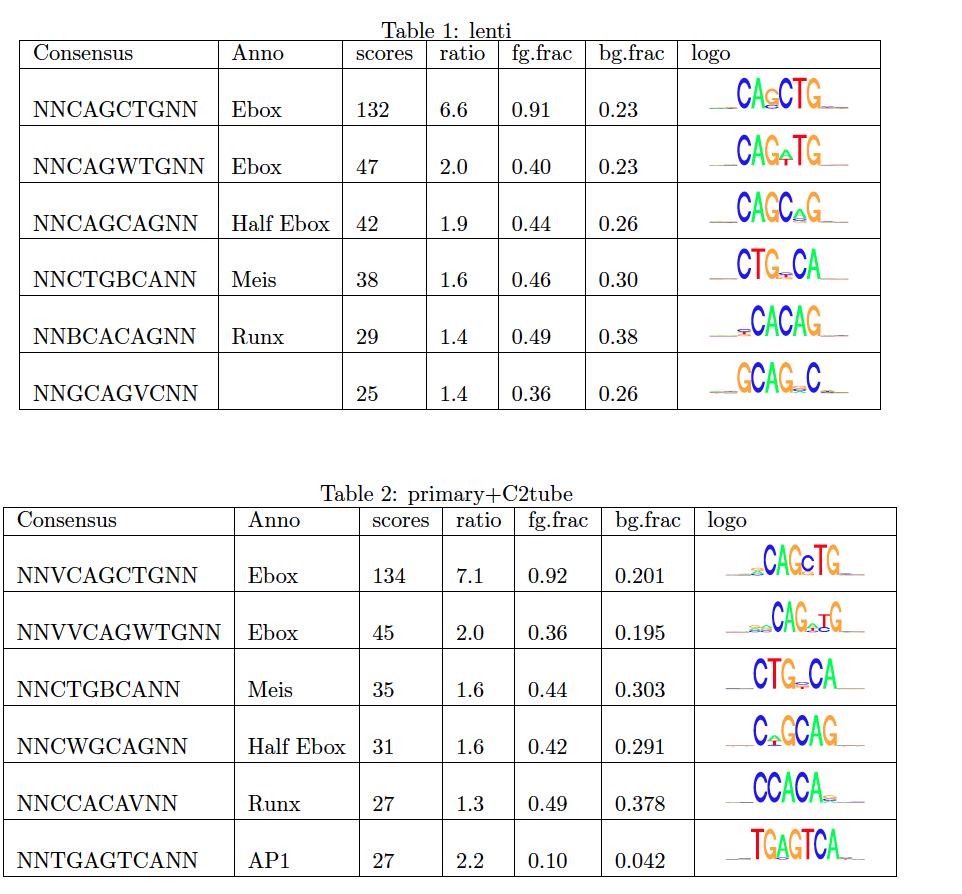


Supplemental Figure 2B.

Discriminative motifs for Lenti specific vs. Primary.tube specific peaks


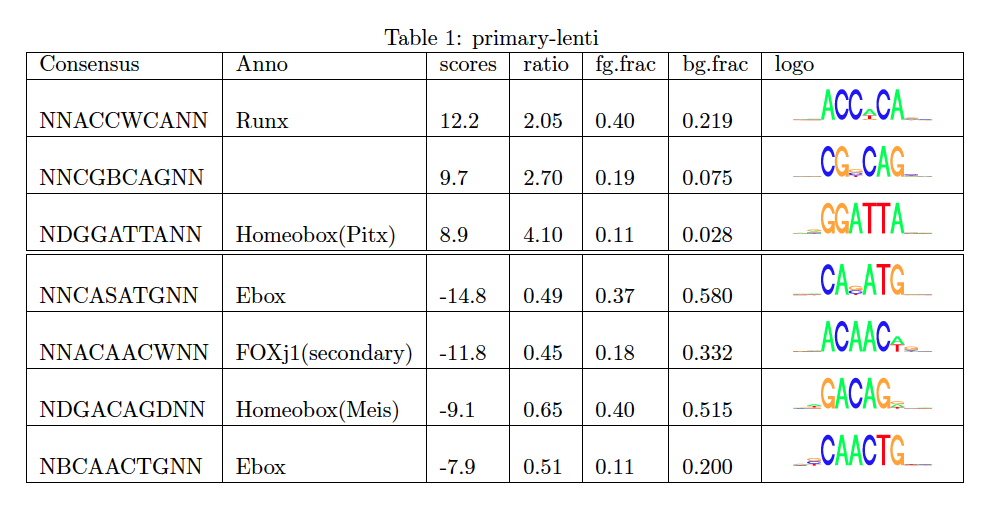

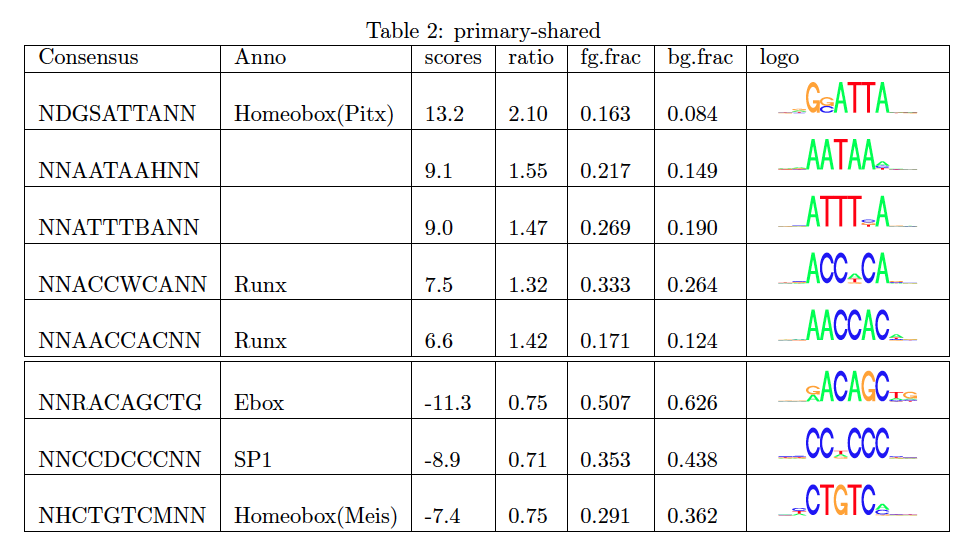


Discriminative motifs for Primary.tube specific peaks vs shared peaks


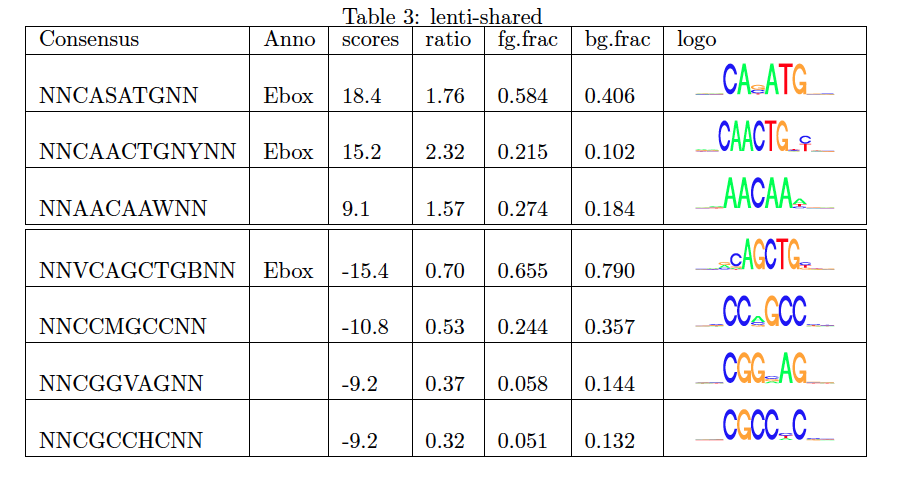


Discriminative motifs for Lenti specific peaks vs shared peaks

**Supplemental Figure S2:** Motif enrichment analysis for regions under over-expressed MyoD peaks and endogenous MyoD peaks. (A) Motifs enriched under all over-expressed MyoD peaks (lenti) or all endogenous MyoD peaks (primary.tube). (B) Motifs specific to endogenous or over-expressed MyoD peaks. Primary-Lenti: Motifs enriched in peaks present only in endogenous MyoD compared to peaks present only in over-expressed MyoD. Primary-Shared: Motifs enriched in endogenous-only peaks compared to peaks present in both groups, i.e., shared peaks. Lenti-Shared: Motifs enriched in peaks only in over-expressed MyoD compared to shared peaks. Consensus, consensus sequence for the motif; Anno, annotated factor for motif consensus; scores, the regression z-values representing the discriminative power of the motif for separating the foreground and background where positive values indicate enriched motifs and negative values indicate depleted motifs; ratio, the enrichment (or depletion) ratio of the motifs in the foreground relative to the background; fg.frac, the percentage of the foreground sequences containing the motif; bg.frac, the percentage of the background sequences containing the motif; logo, the PWM logo.
